# Staged hybrid repair under local anesthesia for ruptured iliac artery aneurysm with iliocaval fistula and acute limb ischemia in a frail patient

**DOI:** 10.1016/j.jvscit.2026.102463

**Published:** 2026-08-22

**Authors:** Won-Gong Chu

**Affiliations:** Division of Vascular Surgery, General Surgery and Cancer Center, Changwon Hanmaeum Hospital, Changwon, Republic of Korea

**Keywords:** Iliocaval fistula, Iliac artery aneurysm, Acute limb ischemia, Staged hybrid management, Endovascular aneurysm repair, Frailty

## Abstract

Iliocaval fistula associated with a ruptured iliac artery aneurysm is rare and life-threatening, particularly when complicated by acute limb ischemia in frail patients. We report the case of a severely frail 78-year-old man with a contained rupture of a common iliac artery aneurysm, iliocaval fistula, Rutherford IIA acute limb ischemia, systemic inflammation, and obstructive uropathy. A staged minimally invasive hybrid strategy under local anesthesia was performed, consisting of uni-iliac endovascular aneurysm repair, delayed femorofemoral bypass, and adjunctive percutaneous nephrostomy for urinary decompression. The patient achieved hemodynamic stabilization, improvement of systemic inflammation, and limb salvage.

Iliocaval fistula is a rare but life-threatening complication of aortoiliac aneurysms, with a reported incidence of less than 1%.^1^ Its presentation is often nonspecific, and diagnosis may be delayed.^1^^,^^2^ When aneurysm rupture is accompanied by acute limb ischemia, systemic inflammation, obstructive uropathy, and severe frailty, treatment requires careful prioritization of fistula exclusion, hemodynamic stabilization, limb salvage, and infection risk.

Open repair has traditionally been considered definitive treatment; however, it can carry substantial morbidity and mortality in frail elderly patients with limited physiologic reserve.^1^^,^^3^ Endovascular repair offers a less invasive alternative for fistula exclusion and hemodynamic stabilization, but adjunctive procedures may be required.^2^^,^^3^ We report a severely frail patient with ruptured common iliac artery aneurysm, iliocaval fistula, and Rutherford IIA acute limb ischemia successfully treated with staged hybrid repair under local anesthesia.

## Case report

A 78-year-old man presented with fever, dyspnea, marked generalized edema involving the lower extremities, upper extremities, and face, and progressive right lower limb ischemia. He had limited mobility requiring wheelchair use due to congenital poliomyelitis and multiple comorbidities, including diabetes mellitus, a history of intracerebral hemorrhage, and early dementia. His Clinical Frailty Scale score was 7, indicating severe frailty.^4^ Written informed consent for publication of this case report and accompanying images was obtained from the patient’s legal representative.

Initial vital signs were relatively stable; however, the overall clinical presentation suggested systemic illness with impaired lower limb perfusion. The right foot was cool and pale, distal pulses were not palpable, and the right ankle-brachial index could not be obtained. Motor function was preserved, sensory deficit was mild, and no fixed skin mottling was observed. Therefore, the limb ischemia was classified as Rutherford IIA acute limb ischemia. Inflammatory markers were elevated, with a C-reactive protein level of 7.17 mg/dL, and further imaging was performed because of the atypical presentation.

**Fig 1:**
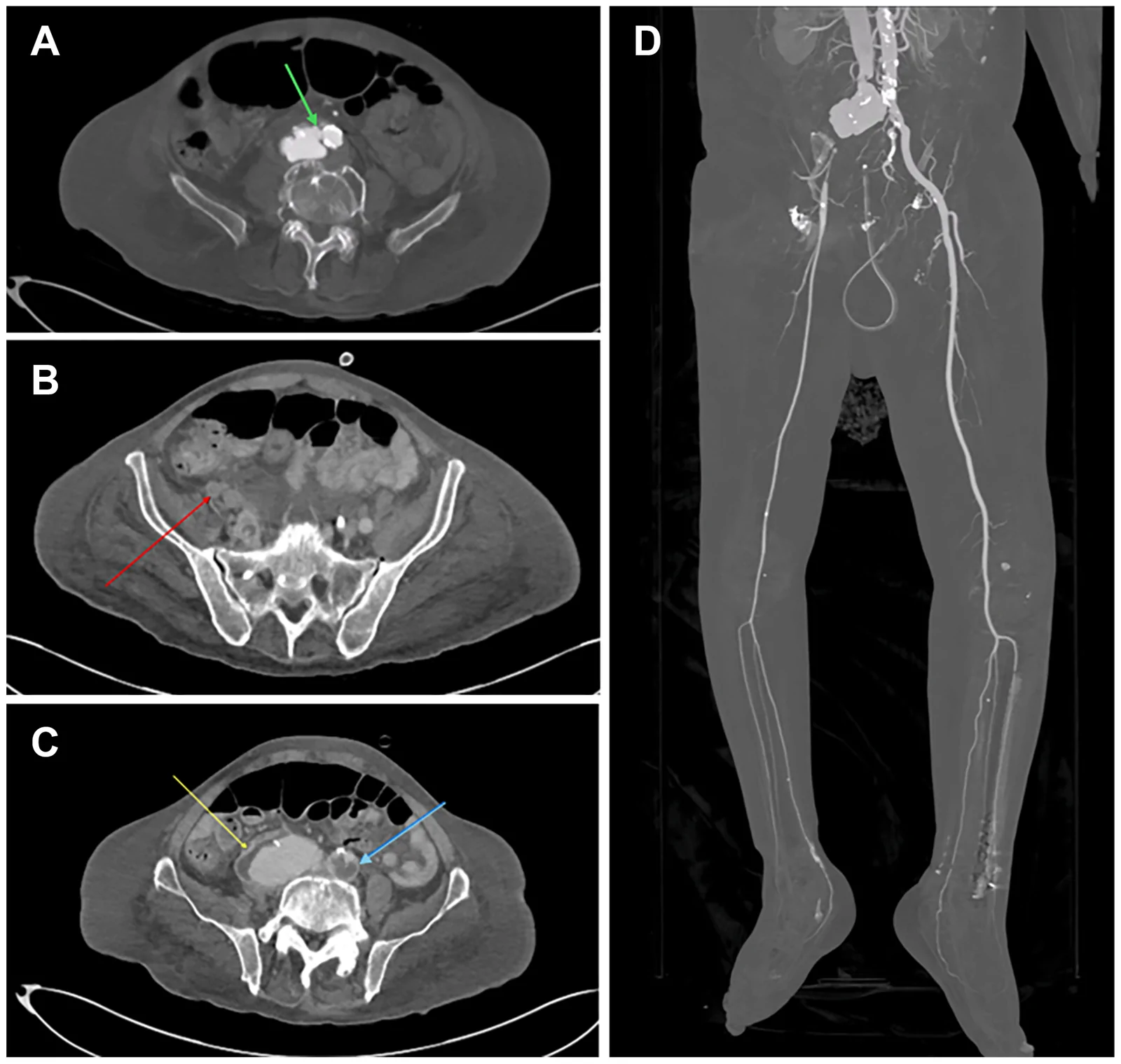
Preoperative computed tomography angiography. **A,** Axial computed tomography image demonstrating a fistulous communication between the aneurysmal common iliac artery and the inferior vena cava, consistent with an iliocaval fistula (*yellow arrow*). **B,** Computed tomography image demonstrating a right common iliac artery aneurysm with mural thrombus and contained rupture. The right external iliac artery is occluded by thrombus (*red arrow*). **C,** Computed tomography image demonstrating degenerative aneurysmal change of the distal left common iliac artery near the iliac bifurcation (*blue arrow*) and a right common iliac artery aneurysm with mural thrombus (*yellow arrow*). Diffuse aneurysmal and atherosclerotic changes are also present. **D,** Maximum intensity projection image demonstrating the overall arterial anatomy and diffuse luminal narrowing of the arterial tree in the right lower extremity.

**Fig 2:**
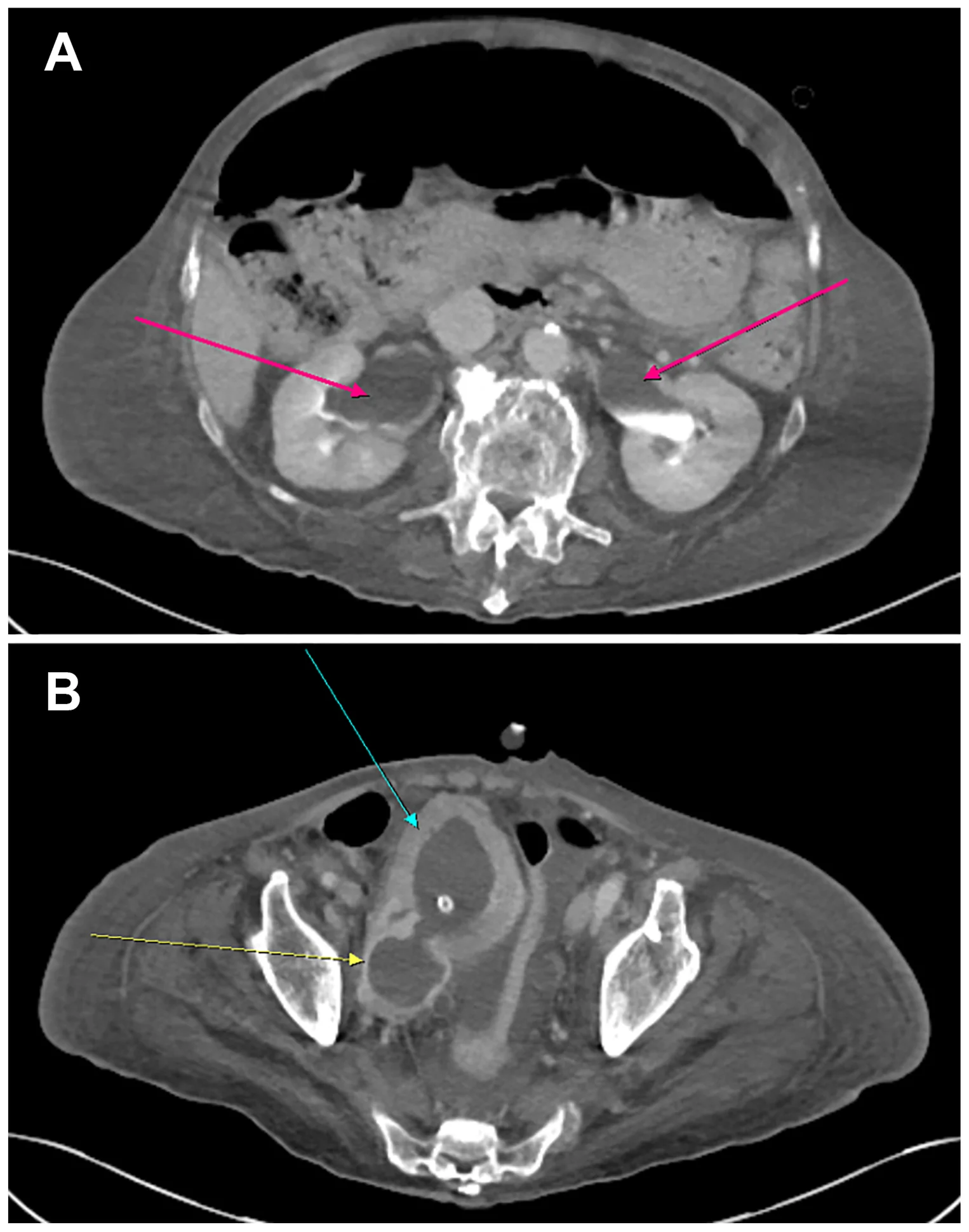
Computed tomography findings suggestive of obstructive uropathy and urinary tract infection. **A,** Venous phase axial computed tomography image showing bilateral hydronephrosis with retained contrast material in the dilated renal collecting systems (*magenta arrows*). **B,** Axial computed tomography image showing bladder wall thickening suggestive of cystitis (*light blue arrow*) and a bladder diverticulum (*yellow arrow*).

Computed tomography (CT) angiography revealed a contained rupture of a common iliac artery aneurysm with an associated iliocaval fistula (Fig 1). The fistulous communication originated from the aneurysmal common iliac artery and drained into the inferior vena cava, consistent with high-flow arteriovenous shunting. Thrombotic occlusion of the right external iliac artery with reduced distal perfusion was also identified and was considered the cause of progressive limb ischemia. The concomitant left-sided aneurysmal lesion involved the left common and internal iliac arteries. Venous-phase axial CT demonstrated bilateral hydronephrosis with contrast retention in the dilated renal collecting systems, consistent with obstructive uropathy from retroperitoneal hematoma-related ureteral compression (Fig 2, *A*).

Initial CT showed no findings suggestive of a mycotic aneurysm, such as sac wall thickening or periaortic inflammatory changes. However, the patient had fever and elevated inflammatory markers, and CT also demonstrated bladder wall thickening suggestive of cystitis (Fig 2, *B*). Therefore, both systemic inflammatory response syndrome associated with the high-flow arteriovenous fistula and possible urinary tract infection related to obstructive uropathy were considered. Blood and urine cultures were obtained on admission, and blood cultures were negative. Empirical antimicrobial therapy consisted of ceftriaxone on hospital day 1 and intravenous meropenem and vancomycin from hospital days 2 to 10. Intravenous fluconazole was initiated after *Candida albicans* was identified in the urine culture. Preoperative echocardiography showed preserved left ventricular systolic function, with a left ventricular ejection fraction of 68.8% and an estimated right ventricular systolic pressure (RVSP) of 41 mmHg.

Given the patient’s severe frailty and high-operative risk, conventional open repair under general anesthesia was considered unsuitable. Retrospective assessment using the American College of Surgeons National Surgical Quality Improvement Program Surgical Risk Calculator estimated a high-perioperative risk, including serious complications of 57.1% and mortality of 25.2%. Therefore, a multidisciplinary decision was made to proceed with a staged minimally invasive hybrid strategy under local anesthesia, with the goals of hemodynamic stabilization, reduction of systemic inflammatory burden, limb salvage, and minimization of graft infection risk.

**Fig 3:**
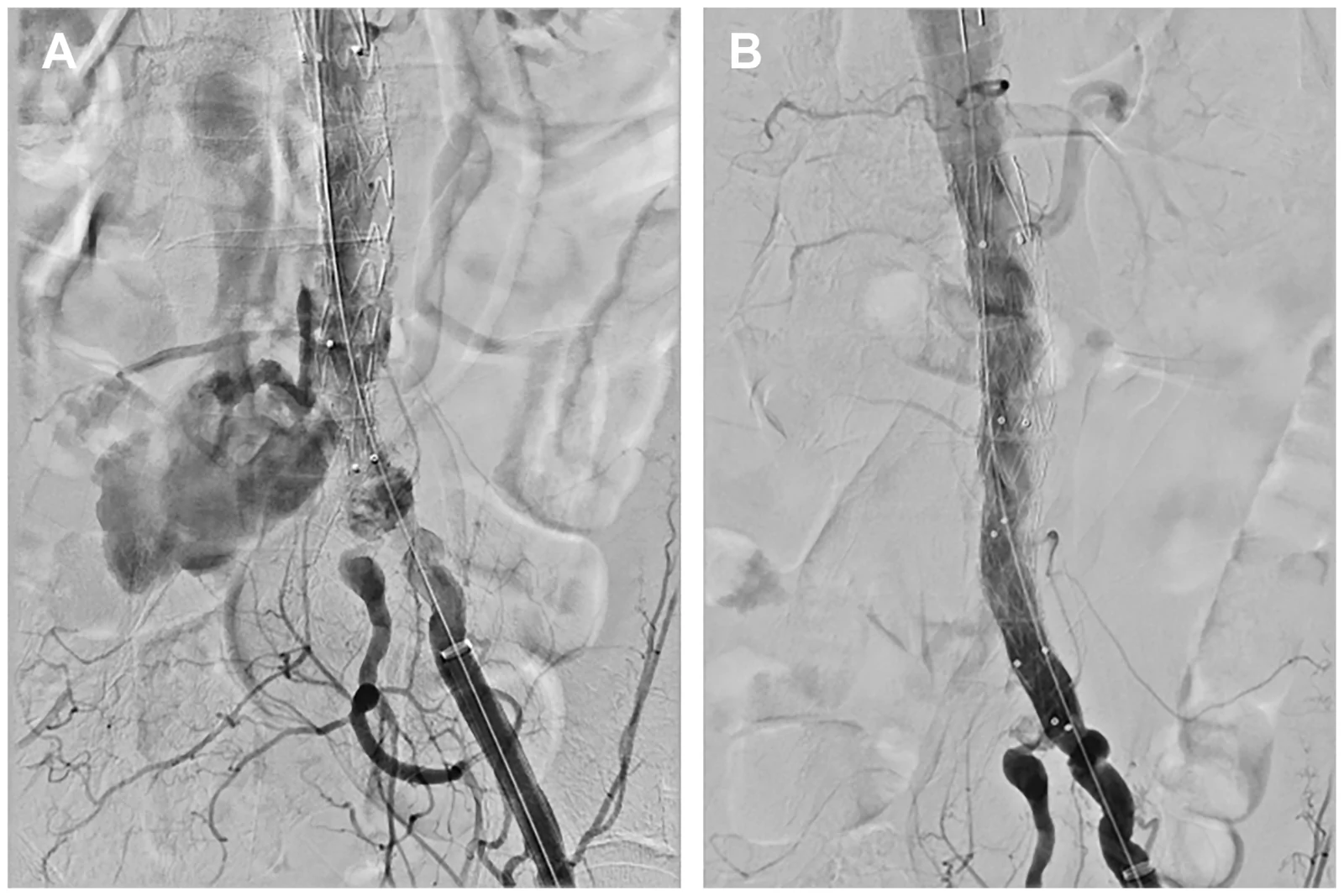
Endovascular aneurysm repair performed under local anesthesia. **A,** Preballoon-molding angiography during uni-iliac endovascular aneurysm repair on hospital day 2 showing the iliac aneurysm with an iliocaval fistula and early opacification of the venous system. **B,** Completion angiography after stent graft deployment demonstrating successful exclusion of the aneurysm and fistula without residual arteriovenous shunting.

As the first step, uni-iliac endovascular aneurysm repair was performed under local anesthesia. The procedure was performed under local infiltration anesthesia using 2% lidocaine, combined with mild intravenous sedation using remifentanil. Completion angiography confirmed successful exclusion of the ruptured aneurysm and iliocaval fistula (Fig 3), allowing initial hemodynamic stabilization and reduction of systemic inflammatory burden. As the right internal iliac artery was already occluded, additional embolization of the left internal iliac artery was not performed to preserve pelvic perfusion. Adjunctive treatment with diuretics and high-flow nasal oxygen was administered, after which dyspnea, pulmonary edema, and generalized edema improved markedly.

On hospital day 3, extra-anatomic femorofemoral bypass was performed to restore perfusion to the ischemic limb. After bypass, the color and warmth of the foot improved, and capillary refill recovered immediately.

The patient also had obstructive uropathy, presumed to be related primarily to the mass effect of the retroperitoneal hematoma. Foley catheterization was performed initially, and percutaneous nephrostomy was subsequently performed on hospital day 6 for urinary decompression and infection control. Urine culture from the nephrostomy output on hospital day 9 yielded *C. albicans* (>10^5^ CFU/mL), for which fluconazole was administered for 1 year. Subsequent cultures yielded vancomycin-resistant *Enterococcus faecium*, for which no targeted therapy was given, and *Klebsiella oxytoca*, which was treated with cefdinir followed by trimethoprim/sulfamethoxazole.

Although no fixed mottling was present at initial presentation, superficial mottled discoloration and digital ischemic changes became apparent on hospital day 3 but subsequently stabilized. Superficial debridement was performed on hospital days 4 and 10. Neither amputation nor additional deep tissue debridement was required, and only routine bedside wound care was continued thereafter.

**Fig 4:**
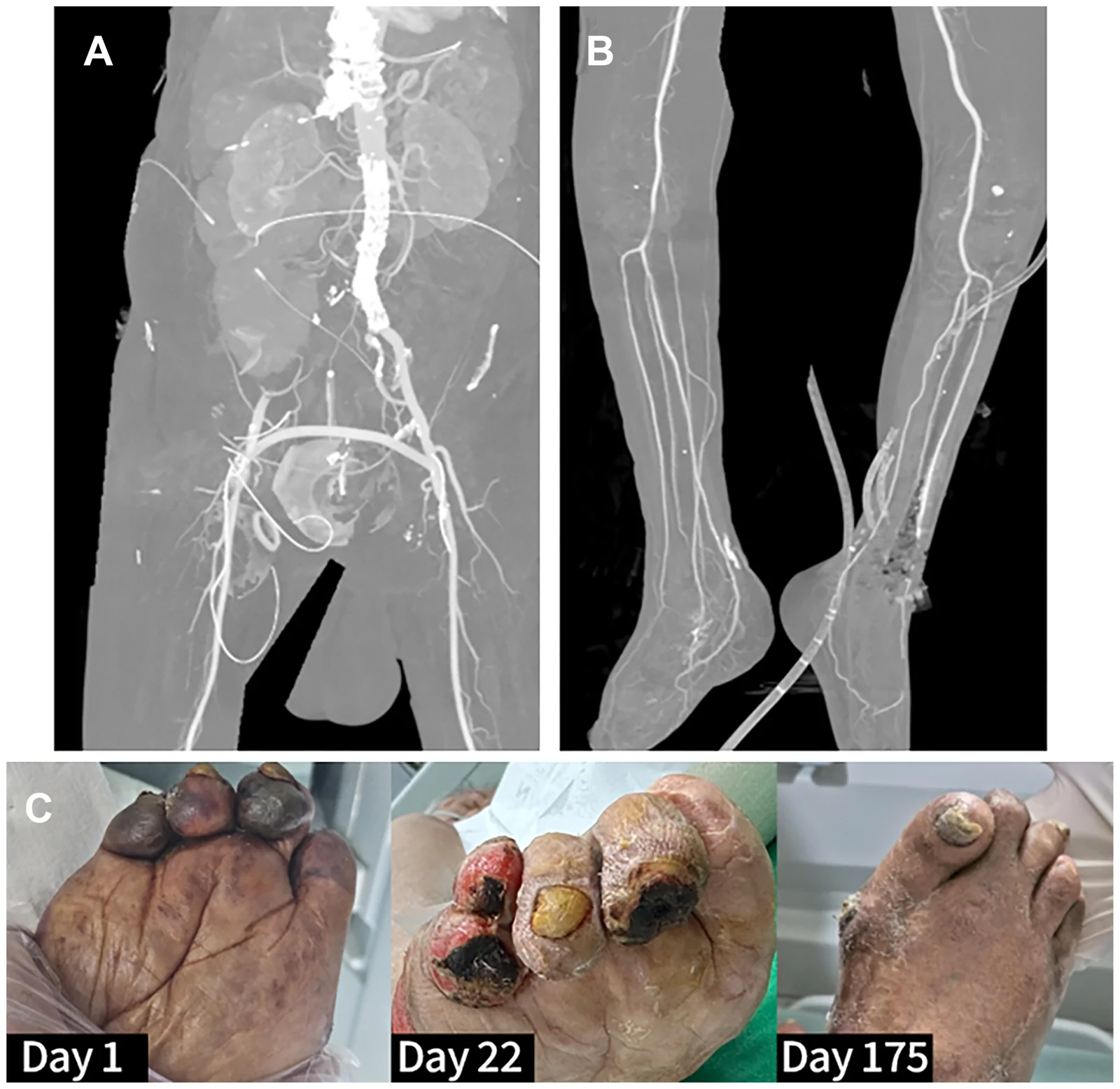
Postoperative outcomes. **A,** Postoperative computed tomography angiography demonstrating successful exclusion of the aneurysm and iliocaval fistula. **B,** Maximum intensity projection image demonstrating restoration of arterial inflow with normalization of arterial caliber in the right lower extremity. **C,** Serial photographs of the right foot demonstrating progressive wound healing from admission through outpatient follow-up. Labels indicate days after admission.

Follow-up imaging confirmed successful exclusion of the aneurysm and iliocaval fistula, as well as adequate restoration of limb perfusion. Serial photographs of the right foot demonstrated progressive wound healing (Fig 4). Inflammatory markers gradually decreased, and no clinical evidence of graft or endograft infection was observed during long-term follow-up. The patient was ultimately discharged on hospital day 26 in stable condition, with preservation of the limb.

## Discussion

Iliocaval fistula associated with ruptured iliac artery aneurysm is a rare but potentially fatal condition, with a reported incidence of less than 1% among aortoiliac aneurysms. Clinical manifestations are highly variable and may include heart failure, abdominal pain, limb ischemia, generalized edema, and systemic inflammatory response.^1^^,^^2^ In the present case, fever, dyspnea, and generalized edema mimicked infection or cardiopulmonary disease. Although cardiac output was not directly quantified, the marked systemic and pulmonary edema, preserved left ventricular ejection fraction, mildly elevated RVSP, and subsequent reductions in left ventricular end-diastolic volume, stroke volume, tricuspid regurgitation velocity, and RVSP after fistula exclusion supported a clinically significant shunt-related high-flow state.

The coexistence of aneurysm rupture, iliocaval fistula, acute limb ischemia, obstructive uropathy, and systemic inflammation presents a major therapeutic challenge. Although open surgical repair has traditionally been regarded as the standard treatment, it carries substantial perioperative morbidity and mortality, particularly in frail patients.^1^ This consideration strongly influenced our decision to avoid extensive open surgery under general anesthesia and instead pursue a minimally invasive strategy.

Endovascular treatment has increasingly been adopted in high-risk patients because of reduced physiologic stress and improved perioperative outcomes.^2^^,^^3^ Previous reports have demonstrated successful endovascular or hybrid treatment of iliocaval or ilioiliac arteriovenous fistulas related to iliac artery aneurysm rupture.^5^, ^6^, ^7^, ^8^, ^9^, ^10^ However, in complex situations such as the present case, a single-stage intervention may not adequately address all competing clinical priorities.

In this patient, a priority-based staged hybrid approach was adopted. Given the risk of potentially fatal rerupture, endograft exclusion was prioritized to promptly eliminate systemic pressure transmission to the ruptured aneurysm sac. Initial endovascular treatment excluded the ruptured aneurysm and iliocaval fistula, reducing high-flow arteriovenous shunting and allowing early hemodynamic stabilization and reduction of systemic inflammatory burden. Subsequent extra-anatomic femorofemoral bypass was then performed as part of a staged revascularization strategy for limb salvage. Importantly, the limb ischemia was classified as Rutherford IIA acute limb ischemia, with preserved motor function and no fixed mottling at presentation. Although concomitant femorofemoral bypass could have enabled earlier limb reperfusion, revascularization was deferred to minimize procedural stress until aneurysm exclusion and clinical stabilization were confirmed. A single-stage approach may be appropriate in selected patients requiring immediate reperfusion.

Infection risk was another important consideration because the patient had diabetes mellitus, severe frailty, obstructive uropathy, candiduria, and retroperitoneal hematoma. The retroperitoneal hematoma was also considered a potential nidus for infection. Accordingly, antibiotics, culture surveillance, urinary drainage, and percutaneous nephrostomy were used to reduce the risk of prosthetic graft or endograft infection.

All major procedures were performed under local anesthesia. This approach minimized physiologic stress while allowing staged treatment in a severely frail patient. Although endovascular or hybrid strategies have been reported previously, fully staged minimally invasive management under local anesthesia remains uncommon.

## Conclusions

A staged hybrid approach under local anesthesia may be a feasible treatment option for selected frail patients with a ruptured iliac artery aneurysm complicated by an iliocaval fistula and acute limb ischemia, minimizing physiologic stress while allowing aneurysm exclusion and subsequent limb revascularization.

## Declaration of generative AI and AI-assisted technologies in the manuscript preparation process

During the preparation of this work, the authors used ChatGPT (OpenAI) to assist with language editing and manuscript structuring. After using this tool, the authors reviewed and edited the content as needed and take full responsibility for the content of the publication.

## Funding

None.

## Disclosures

None.
